# Treatment‐resistant schizophrenia with 22q11.2 deletion and additional genetic defects

**DOI:** 10.1002/npr2.12477

**Published:** 2024-08-27

**Authors:** Sawako Furukawa, Shusei Arafuka, Hidekazu Kato, Tomoo Ogi, Norio Ozaki, Masashi Ikeda, Itaru Kushima

**Affiliations:** ^1^ Department of Psychiatry Nagoya University Graduate School of Medicine Nagoya Japan; ^2^ Department of Psychiatry for Parents and Children Nagoya University Hospital Nagoya Japan; ^3^ Department of Genetics Research Institute of Environmental Medicine (RIeM), Nagoya University Nagoya Japan; ^4^ Pathophysiology of Mental Disorders Nagoya University Graduate School of Medicine Nagoya Japan; ^5^ Medical Genomics Center Nagoya University Hospital Nagoya Japan

**Keywords:** 22q11.2 deletion syndrome, genetics: Human, schizophrenia: Clinical, treatment‐resistant schizophrenia, whole‐genome sequencing

## Abstract

We report a case of a 61‐year‐old female with 22q11.2 deletion syndrome (22q11.2DS) and a novel heterozygous nonsense variant in *MAP1A*, identified through whole‐genome sequencing (WGS). The patient presented with intellectual developmental disorder, treatment‐resistant schizophrenia (SCZ), and multiple congenital anomalies. Despite aggressive pharmacotherapy, she experienced persistent auditory hallucinations and negative symptoms. WGS revealed a 3 Mb deletion at 22q11.2 and a nonsense variant in *MAP1A* (c.4652T>G, p.Leu1551*). *MAP1A*, encoding microtubule‐associated protein 1A, is crucial for axon and dendrite development and has been implicated in autism spectrum disorder and SCZ. The *MAP1A* variant may contribute to the severe psychiatric phenotype, as it is thought to influence synaptic plasticity, a process also affected by 22q11.2 deletion. This case highlights the importance of WGS in identifying additional pathogenic variants that may explain phenotypic variability in 22q11.2DS. Thus, WGS can lead to a better understanding of the genetic architecture of 22q11.2DS. However, further studies are needed to elucidate the role of secondary genetic contributors in the diverse clinical presentations of 22q11.2DS.

## Abstract 2

We report a 61‐year‐old female with 22q11.2 deletion syndrome (22q11.2DS) and a novel heterozygous nonsense variant in MAP1A, identified through whole‐genome sequencing (WGS), who presented with intellectual developmental disorder, treatment‐resistant schizophrenia, and multiple congenital anomalies. The MAP1A variant, which is crucial for axon and dendrite development and has been implicated in autism spectrum disorder and schizophrenia, may contribute to the severe psychiatric phenotype by influencing synaptic plasticity, a process also affected by the 22q11.2 deletion. This case highlights the importance of WGS in identifying additional pathogenic variants that may explain phenotypic variability in 22q11.2DS and underscores the need for further studies to elucidate the role of secondary genetic contributors in the diverse clinical presentations of 22q11.2DS.

## Graphical abstract

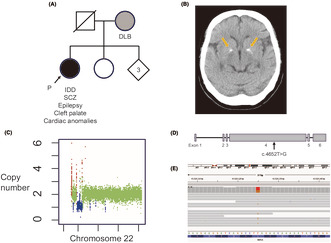

## 1 INTRODUCTION

22q11.2 deletion syndrome (22q11.2DS) is the most prevalent chromosomal microdeletion disorder, with an estimated occurrence of one in 3000–4000 live births, primarily resulting from nonallelic homologous recombination.^1^ The main clinical features of 22q11.2DS include congenital heart disease, palatal anomalies, immune deficiencies, distinctive facial characteristics, and psychiatric disorders.^1^ Notably, 22q11.2DS is recognized as one of the most potent genetic risk factors for schizophrenia (SCZ).^2^ Intellectual developmental disorder (IDD), autism spectrum disorder (ASD), attention deficit/hyperactivity disorder (ADHD), or mood disorders are frequently observed in patients with 22q11.2DS.^2^

The 22q11.2 deletion region contains genes involved in neurodevelopment, several of which play crucial roles in synaptic function.^3^ In line with this, abnormalities in synaptic function, including impaired dendrite and spine development, have been observed in a mouse model of 22q11.2DS.^4^ These synaptic dysfunctions are thought to disrupt the neural circuitry between the hippocampus and prefrontal cortex.^5^ Thus, synaptic and circuit‐level disturbances may play a crucial role in the pathogenesis of psychiatric symptoms and cognitive deficits associated with 22q11.2 deletion.

The psychiatric manifestations and symptom severity in 22q11.2DS exhibit remarkable variability among affected individuals, and the penetrance of the deletion is incomplete.^2^, ^6^ While some patients may present with severe psychotic symptoms and develop SCZ, others may exhibit milder cognitive and behavioral impairments. This phenotypic variability and incomplete penetrance indicate that 22q11.2 deletion alone is not sufficient to explain the diverse clinical outcomes fully, and additional genetic factors likely contribute to the variable expressivity observed in 22q11.2DS. Rare genetic variants outside the 22q11.2 deletion region may contribute to the variability in psychiatric symptom expression in 22q11.2DS,^7^ which indicates that these second‐hit variants that affect genes related to synaptic function and neurodevelopment may modulate the risk of SCZ in individuals with 22q11.2DS. These findings highlight the potential role of genetic factors beyond the primary 22q11.2 deletion in shaping the phenotypic heterogeneity observed in this syndrome.

In this case report, we present a patient with treatment‐resistant SCZ who carried the 22q11.2 deletion along with a psychiatric disorder‐related variant (a nonsense variant in the *MAP1A* gene). MAP1A is involved in synaptic plasticity,^8^ and the combination of 22q11.2 deletion and the *MAP1A* variant may have had an additive effect, resulting in severe psychiatric symptoms in this patient.

## 2 CASE PRESENTATION

**FIGURE 1:**
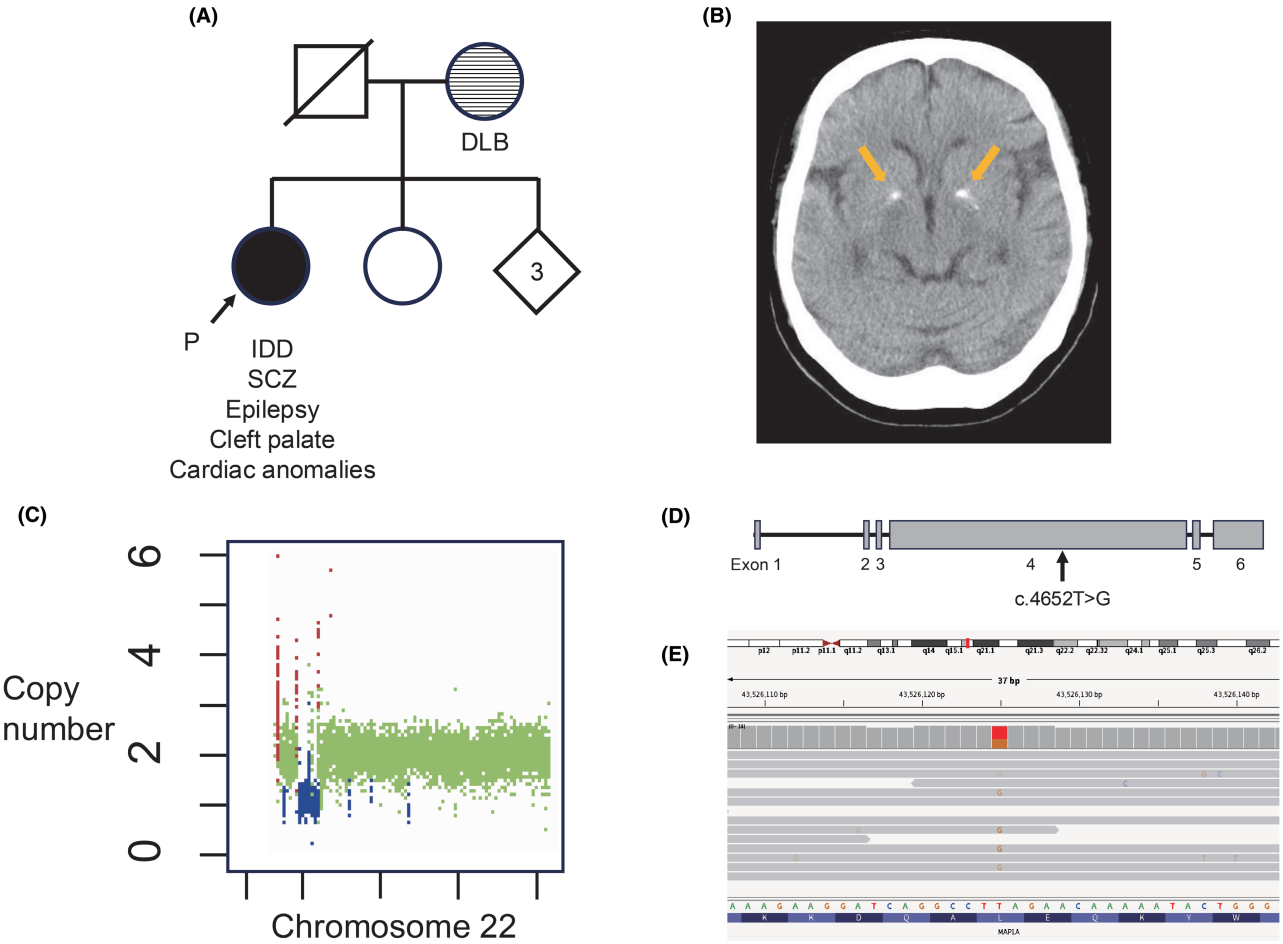
Clinical and genetic characterization of the patient. A) Pedigree of the patient's family. The proband is indicated by an arrow. Filled symbols represent affected individuals, and open symbols represent unaffected individuals. DLB, Dementia with Lewy bodies; IDD, Intellectual developmental disorder; SCZ, Schizophrenia. (B) Brain computed tomography image from when the patient was age 59 years. (C) The 22q11.2 deletion identified by Control‐FREEC. The deletion is shown in blue. (D) Schematic representation of the exon structure of *MAP1A*. (E) Integrative Genomics Viewer snapshot of the *MAP1A* nonsense variant.

The patient was a 61‐year‐old female with moderate IDD and SCZ. Her medical history included ventricular septal defect, atrial septal defect, dysmorphic features, cleft palate, unspecified hernia, epilepsy with yearly generalized tonic–clonic seizures, hypocalcemia, allergic dermatitis, and dyslipidemia. She did not have significant immune dysfunction. Her mother had dementia with Lewy bodies, no other family member had psychiatric symptoms (Figure 1A). The patient was born as the first child among five siblings to parents who were both 23 years old at the time of her birth. A detailed developmental history could not be obtained. She was diagnosed with IDD at age 8 years and attended a special needs school. At age 12 years, she underwent surgical closure for atrial and ventricular septal defects. She had been free from symptoms of heart failure up to the time of this report. After graduation, she worked in cleaning or factory roles. She married at age 32 years but divorced a year later without having children. At age 36 years, she began experiencing hallucinations and delusional persecution, leading to a diagnosis of SCZ. She was hospitalized in a psychiatric ward and experienced auditory hallucinations in which a young man hurled abusive language at her even after hospitalization. Influenced by these auditory hallucinations, she experienced a constantly unstable mood and high irritability. She persistently engaged in impulsive behaviors such as breaking glass and overturning tables. She had delusions that the person appearing in her auditory hallucinations was after her assets. She experienced suicidal thoughts because of these auditory hallucinations and engaged in self‐harm, cutting her own arms. Soliloquy was prominently observed. In her 40s, she was prescribed haloperidol (9 mg), risperidone (3 mg), chlorpromazine (100 mg), olanzapine (10 mg), levomepromazine (50 mg), clonazepam (3 mg), and carbamazepine (600 mg). The chlorpromazine equivalent dose of antipsychotics was 1300 mg. Anticonvulsants were prescribed for not only the management of epilepsy but also their mood‐stabilizing effects. Despite this aggressive pharmacological intervention with multiple antipsychotics, her positive symptoms persisted, leading to a diagnosis of treatment‐resistant SCZ (see the Supporting information).^9^ Over a 20‐year period, she was hospitalized more than 10 times. During her hospitalizations, she frequently felt agitated and called out, which led to complaints from other patients. In recent years, due to negative symptoms, she has shown little interest in her surroundings and has been leading a withdrawn lifestyle. At age 60 years, she was hospitalized in a general hospital because of tetany, convulsions, and diagnosed hypocalcemia. At the time, she had concurrent cellulitis as a spreading infection that triggered hypocalcemia. Medications at age 61 years were olanzapine (10 mg), risperidone (2 mg), and clonazepam (3 mg). Despite this treatment, she experienced persistent auditory hallucinations. Brain computed tomography performed at age 59 years showed bilateral basal ganglia calcifications (Figure 1B).

To investigate the underlying factors contributing to treatment‐resistant SCZ, we performed whole‐genome sequencing (WGS) (Details are provided in the Supporting information). As a result, a 22q11.2 deletion (hg38 chr22:18897001‐21131000) was detected (Figure 1C). Thus, the patient was diagnosed with 22q11.2DS. In addition, a novel heterozygous nonsense variant in exon 4 of *MAP1A* was detected [NM_002373: c.4652T>G, p.Leu1551*; hg38 chr15:g.43526125T>G] (Figure 1D,E). *MAP1A* is highly conserved, with a probability of being loss‐of‐function intolerant score of 1. This variant is located on the middle of the gene (Figure 1D), resulting in the production of truncated MAP1A protein. No other pathogenic variants, including STRs, were detected in this patient.

## 3 DISCUSSION

In this report, we presented a case of a 61‐year‐old female with IDD, SCZ, and multiple comorbidities who exhibited treatment‐resistant positive symptoms despite aggressive pharmacotherapy, frequent psychiatric hospitalizations, and negative symptoms. As mentioned above, patients with 22q11.2DS exhibit notable phenotypic variability.^2^, ^6^, ^7^, ^10^ If records from early childhood were available, a diagnosis of ASD and/or ADHD might have been made. The patient exhibited high irritability, which may have been attributable to her underlying sensory hypersensitivities,^11^ leading to increased distress and agitation.

In addition to genetic factors, environmental factors may also contribute to the phenotypic variability in 22q11.2DS.^12^ To the best of our knowledge, no clear environmental factors, such as poverty, abuse, or bullying, were identified. However, it is conceivable that the patient's experience of divorce before the onset of positive symptoms could have contributed to the development of SCZ, as stressful life events are known to be associated with an increased risk of psychosis.^13^

While there is no evidence for association between 22q11.2DS and treatment resistance, a recent study has reported an increased prevalence of pathogenic CNVs in individuals with treatment‐resistant psychosis compared to those with treatment‐responsive psychosis.^14^ Moreover, we previously reported more severe phenotypes in patients with two pathogenic CNVs.^15^ Based on these findings, we hypothesized that additional genetic factors might have contributed to the development of treatment resistance in this case.

We identified a nonsense variant in *MAP1A* by WGS in addition to 22q11.2DS. Large‐scale whole‐exome sequencing studies have reported that the *MAP1A* nonsense variant is strongly associated with ASD.^16^, ^17^ Microtubule‐associated protein (MAP) 1A, the protein coded by *MAP1A*, is predominantly expressed in neurons and crucial for the formation and development of axons and dendrites.^18^ MAP1A interacts with postsynaptic density protein PSD‐95^19^ and DISC1 (Disrupted‐In‐Schizophrenia 1).^20^ MAP1A anchors *N*‐methyl‐d‐aspartate receptors to the cytoskeleton, stabilizes postsynaptic density scaffolds, and plays a crucial role in the maintenance of synaptic plasticity in the postsynapse.^8^ The mouse model of 22q11.2DS presents abnormal synaptic plasticity in the presynapse.^4^ Therefore, the 22q11.2 deletion and nonsense variants in *MAP1A* are both thought to influence synaptic plasticity, contributing to the severe phenotype observed in this patient. However, the inheritance pattern of the *MAP1A* variant was unknown, the extent to which this variant has influenced the patient's phenotype remains unclear, and further studies are needed to elucidate the role of secondary genetic hits in modulating the clinical presentation of individuals with 22q11.2DS.

In conclusion, we identified a 22q11.2 deletion and a nonsense variant in *MAP1A* in a patient with treatment‐resistant SCZ. These findings indicate that WGS is crucial for gaining a deeper understanding of the genetic architecture of treatment‐resistant SCZ. Furthermore, a nonsense variant in *MAP1A* was detected, which might explain the severe psychiatric phenotype. By utilizing WGS, it is possible to examine patients with 22q11.2DS in greater detail, potentially leading to a more comprehensive understanding of the patient's pathology and treatment options.

## AUTHOR CONTRIBUTIONS

S.F. and I.K. designed the study. S.F. and I.K. performed the genetic analysis. S.F., I.K., S.A., H.K., and N.O. recruited the participants and/or collected DNA samples or phenotype data. S.F. and I.K. wrote the first draft of the manuscript, and the other authors commented on and refined the manuscript. All authors carefully read the manuscript and approved the final version for submission.

## FUNDING INFORMATION

This research was supported by research grants from the Ministry of Education, Culture, Sports, Science and Technology of Japan (MEXT) and the Ministry of Health, Labour and Welfare of Japan, the Japan Agency for Medical Research and Development (AMED) under grant nos. JP20dm0107087, JP21wm0425007, JP21dm0207075, JP21ak0101113, JP21dk0307075, JP20dk0307081, JP21dk0307103, JP21ek0109488, JP21km0405216, JP21ek0109411, JP23ek0109678, and JP22tm0424222, the Japan Society for the Promotion of Science (JSPS) KAKENHI Grant Nos. 23KJ1112, 17H05090, 18H04040, 21K07543, 21H00194, 21H04815, 18K07590, and 15K19720, the CIBoG WISE program from MEXT, and the SENSHIN Medical Research Foundation.

## CONFLICT OF INTEREST STATEMENT

S.F., S.A., H.K., T.O., and I.K. declare no conflicts of interest. N.O. has received research support or speakers' honoraria from, or has served as a consultant to, Sumitomo Dainippon, Eisai, Otsuka, KAITEKI, Mitsubishi Tanabe, Shionogi, Eli Lilly, Mochida, DAIICHI SANKYO, Nihon Medi‐Physics, Takeda, Meiji Seika Pharma, EA Pharma, Pfizer, MSD, Lundbeck Japan, Tsumura, Novartis, Boehringer Ingelheim, Viatris, Kyowa, Janssen, Yoshitomi Yakuhin, Kyowa Kirin, Ono, Astellas, UCB, Taisho Toyama, Medical Review, and Woolsey, outside the submitted work. M.I. has received speakers' honoraria from Sumitomo Pharma, Eisai, Otsuka, Tanabe Mitsubishi, Mochida, Takeda, Meiji Seika Pharma, EA Pharma, Viatris, MSD, Janssen, Lundbeck, Yoshitomi.

## ETHICS STATEMENT

Approval of the Research Protocol by an Institutional Reviewer Board: This study was approved by the ethics committee of Nagoya University Graduate School of Medicine (2010‐1033). This study complied with all the provisions of the Declaration of Helsinki.

Informed consent: Written informed consent was obtained from the patient.

Registry and the Registration No. of the Study/Trial: N/A.

Animal Studies: N/A.

## Supporting information


Data S1.

## Data Availability

The data that support the findings of this study are available in this article and its supporting information files.

## References

[1] McDonald‐McGinn DM , Sullivan KE , Marino B , Philip N , Swillen A , Vorstman JA , et al. 22q11.2 deletion syndrome. Nat Rev Dis Primers. 2015;1:15071. 27189754 10.1038/nrdp.2015.71 PMC4900471

[2] Schneider M , Debbane M , Bassett AS , Chow EW , Fung WL , van den Bree M , et al. Psychiatric disorders from childhood to adulthood in 22q11.2 deletion syndrome: results from the International Consortium on Brain and Behavior in 22q11.2 Deletion Syndrome. Am J Psychiatry. 2014;171(6):627–639. 24577245 10.1176/appi.ajp.2013.13070864 PMC4285461

[3] Nehme R , Pietilainen O , Artomov M , Tegtmeyer M , Valakh V , Lehtonen L , et al. The 22q11.2 region regulates presynaptic gene‐products linked to schizophrenia. Nat Commun. 2022;13(1):3690. 35760976 10.1038/s41467-022-31436-8 PMC9237031

[4] Earls LR , Bayazitov IT , Fricke RG , Berry RB , Illingworth E , Mittleman G , et al. Dysregulation of presynaptic calcium and synaptic plasticity in a mouse model of 22q11 deletion syndrome. J Neurosci. 2010;30(47):15843–15855. 21106823 10.1523/JNEUROSCI.1425-10.2010 PMC3073555

[5] Karayiorgou M , Simon TJ , Gogos JA . 22q11.2 microdeletions: linking DNA structural variation to brain dysfunction and schizophrenia. Nat Rev Neurosci. 2010;11(6):402–416. 20485365 10.1038/nrn2841 PMC2977984

[6] Nakatochi M , Kushima I , Ozaki N . Implications of germline copy‐number variations in psychiatric disorders: review of large‐scale genetic studies. J Hum Genet. 2021;66(1):25–37. 32958875 10.1038/s10038-020-00838-1

[7] Bassett AS , Lowther C , Merico D , Costain G , Chow EWC , van Amelsvoort T , et al. Rare genome‐wide copy number variation and expression of schizophrenia in 22q11.2 deletion syndrome. Am J Psychiatry. 2017;174(11):1054–1063. 28750581 10.1176/appi.ajp.2017.16121417 PMC5665703

[8] Takei Y , Kikkawa YS , Atapour N , Hensch TK , Hirokawa N . Defects in synaptic plasticity, reduced NMDA‐receptor transport, and instability of postsynaptic density proteins in mice lacking microtubule‐associated protein 1A. J Neurosci. 2015;35(47):15539–15554. 26609151 10.1523/JNEUROSCI.2671-15.2015 PMC6705472

[9] Howes OD , McCutcheon R , Agid O , de Bartolomeis A , van Beveren NJ , Birnbaum ML , et al. Treatment‐resistant schizophrenia: Treatment Response and Resistance in Psychosis (TRRIP) Working Group consensus guidelines on diagnosis and terminology. Am J Psychiatry. 2017;174(3):216–229. 27919182 10.1176/appi.ajp.2016.16050503 PMC6231547

[10] Toyosima M , Maekawa M , Toyota T , Iwayama Y , Arai M , Ichikawa T , et al. Schizophrenia with the 22q11.2 deletion and additional genetic defects: case history. Br J Psychiatry. 2011;199(3):245–246. 21881099 10.1192/bjp.bp.111.093849

[11] Robertson CE , Baron‐Cohen S . Sensory perception in autism. Nat Rev Neurosci. 2017;18(11):671–684. 28951611 10.1038/nrn.2017.112

[12] Hiroi N , Takahashi T , Hishimoto A , Izumi T , Boku S , Hiramoto T . Copy number variation at 22q11.2: from rare variants to common mechanisms of developmental neuropsychiatric disorders. Mol Psychiatry. 2013;18(11):1153–1165. 23917946 10.1038/mp.2013.92 PMC3852900

[13] Modasi J , Khachadourian V , O'Hora K , Kushan L , Slavich GM , Shields GS , et al. Associations between acute and chronic lifetime stressors and psychosis‐risk symptoms in individuals with 22q11.2 copy number variants. Psychol Med. 2023;53(15):7222–7231. 37078394 10.1017/S0033291723000740 PMC10719673

[14] Farrell M , Dietterich TE , Harner MK , Bruno LM , Filmyer DM , Shaughnessy RA , et al. Increased prevalence of rare copy number variants in treatment‐resistant psychosis. Schizophr Bull. 2023;49(4):881–892. 36454006 10.1093/schbul/sbac175 PMC10318882

[15] Kushima I , Aleksic B , Nakatochi M , Shimamura T , Shiino T , Yoshimi A , et al. High‐resolution copy number variation analysis of schizophrenia in Japan. Mol Psychiatry. 2017;22(3):430–440. 27240532 10.1038/mp.2016.88

[16] Satterstrom FK , Walters RK , Singh T , Wigdor EM , Lescai F , Demontis D , et al. Autism spectrum disorder and attention deficit hyperactivity disorder have a similar burden of rare protein‐truncating variants. Nat Neurosci. 2019;22(12):1961–1965. 31768057 10.1038/s41593-019-0527-8 PMC6884695

[17] Satterstrom FK , Kosmicki JA , Wang J , Breen MS , De Rubeis S , An JY , et al. Large‐scale exome sequencing study implicates both developmental and functional changes in the neurobiology of autism. Cell. 2020;180(3):568–584. e23. 31981491 10.1016/j.cell.2019.12.036 PMC7250485

[18] Halpain S , Dehmelt L . The MAP1 family of microtubule‐associated proteins. Genome Biol. 2006;7(6):224. 16938900 10.1186/gb-2006-7-6-224 PMC1779536

[19] Reese ML , Dakoji S , Bredt DS , Dotsch V . The guanylate kinase domain of the MAGUK PSD‐95 binds dynamically to a conserved motif in MAP1a. Nat Struct Mol Biol. 2007;14(2):155–163. 17220895 10.1038/nsmb1195

[20] Morris JA , Kandpal G , Ma L , Austin CP . DISC1 (disrupted‐in‐schizophrenia 1) is a centrosome‐associated protein that interacts with MAP1A, MIPT3, ATF4/5 and NUDEL: regulation and loss of interaction with mutation. Hum Mol Genet. 2003;12(13):1591–1608. 12812986 10.1093/hmg/ddg162

