# Supplementary material for: Treatment‐resistant schizophrenia with 22q11.2 deletion and additional genetic defects
Source: Neuropsychopharmacol Rep. 2024 Aug 27;44(4):847–51. doi: 10.1002/npr2.12477 (PMC11609749; doi:10.1002/npr2.12477)
Supplement: Supplementary file 1 — Data S1. [file NPR2-44-847-s001.docx]

**SUPPLEMENTARY INFORMATION**

**Treatment-resistant schizophrenia with 22q11.2 deletion and additional genetic defects**

Sawako Furukawa^1^, Shusei Arafuka^1^, Hidekazu Kato^1,2^, Tomoo Ogi^3^, Norio Ozaki^4^, Masashi Ikeda^1^, Itaru Kushima*^1,5^

**Details of Genetic Analysis**

Genomic DNA was extracted from blood samples using a Qiagen QIAamp DNA blood kit (Qiagen, Hilden, Germany). An MGIEasy FS DNA Prep kit (MGI, Shenzhen, China) was used for library construction. Sequencing was performed on MGI’s DNBSEQ-T7 platforms in 150-bp paired-end mode to an average depth of 30×. We used the NVIDIA Parabricks pipeline for alignment and variant calling (10), which accelerates bwa-mem (https://github.com/lh3/bwa) and the Genome Analysis Toolkit Best Practice pipeline (https://gatk.broadinstitute.org/hc/en-us) software on a GPU. Single nucleotide variants and insertions/deletions called by the Parabricks pipeline were annotated by ANNOVAR (11). We excluded intergenic variants and variants with a minor allele frequency $\geq$1% of the genomes in the East Asian populations of the gnomAD database and the Tohoku Medical Megabank Organization. For large CNVs, we applied Control-FREEC (12) and ERDS (13). For other structural variants (SVs) including small CNVs, we used Manta (14). AnnotSV was used to annotate CNV/SV calls (15), with common CNVs/SVs excluded. ExpansionHunter was used to detect known pathogenic short tandem repeats (STRs) (16).

**Evaluation of Treatment Resistance**

Treatment resistance was evaluated retrospectively using the criteria of Treatment-Response and Resistance in Psychosis (TRRIP) Working Group Consensus Guidelines (1). The criteria included: 1) At least moderate severity and <20% symptom reduction during treatment of ≥6 weeks; 2) ≥12 weeks of treatment resistance; 3) ≥6 weeks at a therapeutic dosage; 4) Equivalent to ≥600 mg of chlorpromazine per day; 5) ≥2 past adequate treatment episodes with different antipsychotic drugs; 6) ≥80% of prescribed doses taken; 7) Positive, negative, and cognitive symptoms were observed.

1. Howes OD, McCutcheon R, Agid O, de Bartolomeis A, van Beveren NJ, Birnbaum ML, et al. Treatment-Resistant Schizophrenia: Treatment Response and Resistance in Psychosis (TRRIP) Working Group Consensus Guidelines on Diagnosis and Terminology. Am J Psychiatry. 2017;174(3):216-29.
